# How does the immune system learn to distinguish between good and evil? The first definitive studies of T cell central tolerance and positive selection

**DOI:** 10.1007/s00251-019-01127-8

**Published:** 2019-08-15

**Authors:** Paweł Kisielow

**Affiliations:** Hirszfeld Institute of Immunology and Experimental Therapy, Polish Academy of Sciences, Rudolf Weigl St. 12, 53-114 Wroclaw, Poland

**Keywords:** T cells, Positive and negative selection, Self/nonself discrimination

## Abstract

Demonstration that immature CD4 + 8+ thymocytes contain T cell precursors that are subjected to positive and negative selection was the major step towards understanding how the adaptive immune system acquires the ability to distinguish foreign or abnormal (mutated or infected) self-cells from normal (healthy) cells. In the present review, the roles of TCR, CD4, CD8, and MHC molecules in intrathymic selection and some of the crucial experiments that contributed to the solution of the great immunological puzzle of self/nonself discrimination are described in an historical perspective. Recently, these experiments were highlighted by the immunological community by awarding the 2016 Novartis Prize for Immunology to Philippa Marrack, John Kappler, and Harald von Boehmer.

## Introduction

The “Good” that is protected by the immune system consists of properly functioning healthy cells, and the “Evil” that is fought against consists of “sick” (i.e., infected or cancerous) self-cells and pathogenic microorganisms. The fundamental question that puzzled researchers since more than a century was: *How can the immune system discriminate between good and evil, and does not turn its deadly arsenal of defence against its own normal cells*?

In 2016, during the last congress of the Immunological Societies in Melbourne (Australia), three immunologists, Harald von Boehmer, Philippa Marrack, and John Kappler, were awarded the prestigious “Novartis Prize for Immunology” for elucidating mechanisms responsible for the acquisition by the immune system of the above-mentioned wonderful ability of self/nonself discrimination. The weight of the problem and the scale of the difficulties that had to be overcome to achieve this success is reflected by the fact that several scientists that conquered these obstacles were honored by the Nobel Prize: Emil von Behring, 1901; Paul Ehrlich, 1908; Macfarlane Burnet, 1959; Peter Medawar, 1960; Gerald Edelman and Roney Porter, 1972; Baruj Benaceraff, Jean Dausset, and George Snell, 1983; Niels Jerne, George Koehler, and Cesar Milstein 1984; Susumu Tonegawa, 1987; Peter Doherty and Rolf Zinkernagel, 1996; Ralph Steinman, 2011.

As a participant in the research that contributed to the elucidation of the great and old immunological puzzle of/self/nonself discrimination, I will describe the road leading to deciphering the unusually demanding and raw process of “education” of pivotal cells of the immune system that forces the majority of its “pupils” to commit suicide.

## Difficult beginnings

The necessity of a mechanism enabling the immune system to distinguish “good” from “evil” was postulated at the beginning of the twentieth century by Paul Ehrlich, the author of the famous expression “horror autotoxicus,” when a seemingly endless variety and unprecedented specificity of antibodies—discovered by Emil von Behring—was demonstrated. The diversity appeared to be so great that organisms in every vertebrate class tested were shown to produce antibodies fitting precisely any, not only nonself but also self proteins, potentially resulting in serious diseases or even death. How could organisms avoid such a danger?

For more than 50 years, no significant progress was made in this area. One did not know which cells produced antibodies and there was no concept to direct the research into the right direction. Particularly puzzling was the origin of the huge diversity of antibodies: according to the knowledge at that time, our genomes literally could not contain enough genes to encode the billions of different antibodies found in each individual.

The breakthrough occurred when Niels Kaj Jerne (Jerne 1955) reintroduced the concept that the antigen, i.e., a molecule that induces an immune response, does not represent the template on which the antibody is formed as postulated earlier by Pauling, but acts as a specific selective factor. Soon, David Talmage and Frank McFarlane Burnet (Talmage 1957; Burnet 1957) formulated a theory providing a cellular context to this concept, the clonal selection theory. James Gowans with Johnathan Uhr (Gowans and Uhr 1966) then showed that some of the small white blood cells, called lymphocytes, that constitute the major population of the cells in the spleen and lymph nodes produce antibodies. In a nutshell, according to Jerne, Talmage and Burnet, from the ocean of cells that can potentially produce the whole spectrum of different antibodies (each single cell makes antibodies of only one specificity), the antigen selects the cell that expresses on its surface the unique antibody that binds the antigen and this interaction induces that cell’s proliferation and differentiation into an antibody-secreting cell. On the other hand, the failure to respond to self molecules called “immunological tolerance” would be the consequence of deletion (negative selection) of lymphocytes that recognized antigen before reaching maturity (Lederberg 1959); this was first suggested in experiments in Medawar’s laboratory demonstrating the induction of transplantation tolerance in adults by neonatal injection of foreign donor cells (Billingham et al.^1953^).

## Golden times: uncovering the diversity of lymphocytes and molecules regulating their activity

The desire to verify this inspiring concept of immunological tolerance was for many years the driving force of investigations that uncovered the huge complexity of the immune system. The discovery that morphologically homogenous lymphocytes are functionally heterogeneous (Claman et al. 1966; Mitchell and Miller 1968; Kisielow et al. 1975) made it clear that the problem was much more complicated than originally thought. It appeared that the functioning of the immune system, including antibody production by B lymphocytes or B cells (so-called because they originate and mature in the bone marrow), is governed by T lymphocytes or T cells (so-called because they mature in the thymus). T cells do not produce antibodies but recognize antigens with the same specificity and precision as B cells, however, in a more complicated fashion (Zinkernagel and Doherty 1974; Kappler et al. 1981; Dembic et al. 1986). As a consequence, researchers concentrated on investigating the “methods of education” of T cells and discovered that their activity is under control of a quintet of surface molecules: TCR, MHC class I, MHC class II, CD4, and CD8, where TCR plays the first violin. Identification of each of these molecules opened a new chapter in contemporary history of immunology. The elucidation of their role in an “educated” immune system helped to formulate how immature T cells develop and learn to perform their craft that demands unusually high selectivity and precision.

## TCR and MHC

The T cell receptor (TCR) (McIntyre and Allison 1983; Haskins et al. 1983; Hedrick et al. 1984; Yanagi et al. 1984) is a cell surface transmembrane molecule that resembles antibody molecule. Both molecules are built from immunoglobulin superfamily domains. The same mechanism that is responsible for the generation of antibody diversity generates the diversity of TCRs and ensures that each T lymphocyte and its progeny (clone) expresses a unique TCR. Genes encoding TCR are assembled in each immature lymphocyte like in a kaleidoscope, by rearrangement of short DNA fragments from three different families, in each case in a unique combination (Hozumi and Tonegawa 1976; Tonegawa 1988). The discovery of this novel mechanism of DNA rearrangement solved the fundamental problem of the ability of DNA to encode essentially an unlimited number of different antibodies and TCRs. Compared to an antibody, the distinctive feature of the TCR is its dual specificity, which is the key to understanding the ability of the immune system to distinguish self-cells that are “healthy” from those that are “sick” or foreign. In contrast to antibodies, TCRs recognize antigens only on the surface of other cells in the form of a complex composed of peptides, i.e., short protein fragments resulting from their intracellular degradation, bound by one of the two kinds of major histocompatibility complex (MHC) molecules: class I and class II. MHC molecules function as conveyor belts ferrying peptides from the inside of the cell to its surface in order to make them visible to T cells. Circulating “educated” T cells scan MHC molecules—frequent interaction with them is necessary for T cell survival (Kisielow and Miazek 1995; Kirberg et al. 1997)—in search of peptides derived from mutated self (e.g., carcinogenic) or foreign (e.g., viral) proteins, which they did not encounter during their development and maturation. The recognition of such peptides activates T cells to carry out their duty and eliminate the threat. Every vertebrate (with some rare exceptions) is endowed with different sets of MHC molecules (Dausset 1981), which constitute its individual “ID card” enabling T cells to discriminate between self and foreign cells.

## CD4 and CD8

The CD4 (Reinherz et al. 1979; Dialynas et al. 1983) and CD8 (Boyse et al. 1968) molecules are generally required as co-receptors of the TCR for T cell activation. They characterize two major, functionally different populations of T cells (Kisielow et al. 1975; Cantor and Boyse 1975a; Cantor and Boyse 1975b; Reinherz et al. 1979): CD4 T cells regulate the activity of other cells involved in the immune response through their production of cytokines and CD8 T cells kill infected or mutated (e.g., cancerous) cells.

T cells that recognize a specific peptide on the surface of other cells cannot be activated unless TCR becomes bridged with CD4 or CD8 molecule by MHC/peptide complexes. Class I molecules cross-link TCR with CD8 molecules and class II molecules cross-link TCR with CD4 molecules. The expression of class I molecules on all cells in the organism ensures that each cell presenting a viral or mutation-specific peptide that was not seen during development in the thymus can be killed by cytotoxic CD8 T cells. In contrast, expression of MHC class II molecules is restricted to subsets of cells of the immune system, thus ensuring that the regulatory activity of CD4 T cells is concentrated on cells that participate in the immune response, without impacting other cells.

## Positive selection

Because of the functional heterogeneity of T cells and the discovery of crucial molecules regulating their activity it became clear that self-antigen induced negative selection alone could not totally account for the education of T cells. It was important to elucidate how “useful” T lymphocytes that can recognize self MHC alleles are saved from genetically programmed cell death (apoptosis) and induced to become CD4 or CD8 T cells. As suggested by the experiments of Bevan (Bevan 1977) and Zinkernagel (Zinkernagel 1978), an active process of positive selection seemed necessary but there was no direct evidence and the mechanism was not known. What was the role of the above-mentioned molecules in these processes? Where and at which stage of maturation did the key events of negative and positive selection occur, shaping the TCR repertoire in such a way that it will react only against non-self and modified self but not against self-antigens?

Answers to these questions were provided by experiments using genetically modified mouse models (von Boehmer and Kisielow 1990; Kisielow and Boehmer 1995).

## The role of the thymus

From the time when it was shown that neonatal thymectomy impairs the immune response (Miller 1961), it was strongly suspected that it is the organ in which T lymphocytes are generated from precursor cells called thymocytes. However, this was very difficult to prove because, for a long time, no tools were available to follow the fate of seemingly identical thymocytes that, as suggested by early experiments, never leave the thymus (McPhee et al. 1979). It could not be excluded that the thymus represents the “cemetery” of “useless” lymphocytes and its influence on the development of T lymphocytes might be indirect, mediated by secreted hormones. This view became untenable when it was shown that a majority of thymocytes express TCR (Snodgrass et al. 1985) and that CD4 and CD8 molecules define three different populations of thymocytes (Kisielow et al. 1975) appearing in the following sequence during intra-thymic development: first double-positive CD4+8+ and then single-positive CD4+8- and CD4-8+ (Ceredig et al. 1983; Kisielow et al. 1984). Such a sequence suggested that the dominating (> 90%) population of non-dividing CD4+8+ thymocytes contains not only dying cells but also precursors of mature single positive CD4 and CD8 T lymphocytes. In order to obtain evidence that this is the case and to determine the developmental stage(s), at which negative and positive selection take place, it was necessary to follow the fate of a single thymocyte equipped with TCR of one defined specificity. However, for many years, reagents were not available to find this “needle in the haystack” and the field languished.

The first result that broke the impasse was obtained in the laboratory of Philippa Marrack and John Kappler (Kappler et al. 1987). Tracing the fate of T lymphocytes expressing TCR with known specificity for a so-called superantigen, they made the observation, consistent with the clonal selection hypothesis, that immunological tolerance results from a negative selection of immature lymphocytes. However, because superantigens are special molecules that bind TCR in a different way than the MHC/peptide complexes, it was not clear whether binding to normal self-antigens (i.e., self peptides associated with MHC) also results in lymphocyte deletion. Moreover, the stage at which developing T cells became deleted could not be precisely identified. The real breakthrough occurred when transgenic animals, expressing the “transplanted” genes encoding TCR of a defined specificity became available for such studies (Kisielow et al. 1988a; Sha et al. 1988).

## HY-TCR transgenic mice

Transgenic mice (Kisielow et al. 1988a) obtained in the laboratory of Harald von Boehmer, in collaboration with several other laboratories, allowed us to trace the development of thymocytes that expressed TCRs with only one specificity for a defined MHC-peptide complex. All thymocytes and T cells in these mice expressed a TCR that recognized the male-specific HY peptide presented by MHC class I molecules of a given allele (D^b^). In males, the TCRs recognized both the HY peptide and D^b^ molecule, and almost all thymocytes were deleted at the double-positive CD4+8+ stage (Kisielow et al. 1988a). In female mice bearing the same class I allele (D^b^), there was no wholesale deletion of thymocytes, and most of the cells were CD8 single positive, showing that they were positively selected on the particular class I allele (Teh et al. 1988; Kisielow et al. 1988b). In female mice expressing different class I allele (D^k^), no clear evidence of negative or positive selection of thymocytes expressing transgenic HY specific TCR was observed: the proportion of transgenic double-positive CD4+8+ thymocytes was similar to proportion of CD4+8+ thymocytes in normal non-transgenic mice and single positive CD8 or CD4 thymocytes expressing transgenic HY specific TCR were not found in significant numbers. Thus, comparison of the fate of developing thymocytes in TCR transgenic males and females allowed us to gain insight into the roles of each of the five key molecules (TCR, CD4, CD8, MHC class II, and MHC class I). These results provided framework for understanding the mechanisms of intrathymic education of T cells that are responsible for their ability to destroy “sick” cells without harming “healthy” ones.

## Cellular mechanisms of negative and positive selection

**Fig. 1:**
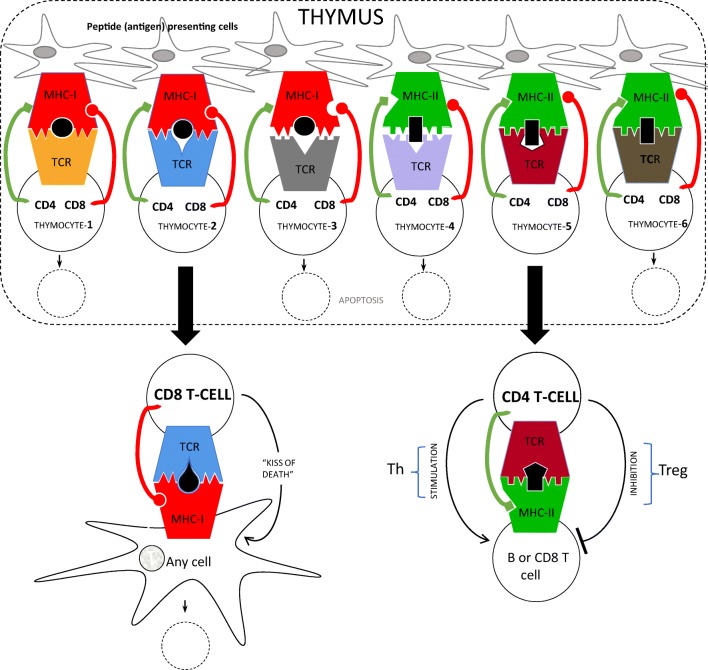
Positive and negative selection in the thymus

The TCR repertoire of mature T cells is shaped in the thymus by two opposite processes of negative and positive selection of CD4+8+ thymocytes (Kisielow et al. 1988a; von Boehmer et al. 1989; Swat et al. 1991; Kaye et al. 1989; Swat et al. 1994, and Fig. 1). Their fate depends mainly on the degree of complementarity between TCR and MHC molecules presenting self-peptides. Cells expressing “useless” TCR, i.e., having no complementarity to self MHC/peptide complex die by spontaneous apoptosis, genetically programmed suicidal cell death (thymocytes 3 and 4; this is the case above with female TCR transgenic thymocytes not matched to the class I allele). This is referred to as “death by neglect”. Cells expressing TCR that are fully complementary for both MHC and presented (cognate) peptide (the case above of male thymocytes recognizing the HY peptide on a particular class I allele), and bind the complex with high affinity, are also negatively selected and die by antigen-induced apoptosis via TCR signaling (thymocytes 1 and 6). This is referred to as “clonal deletion” or “central tolerance.” Cells bearing a TCR matching a particular MHC allele not bound to the cognate peptide, are “saved,” i.e., positively selected and protected from death by neglect, and their further development depends on the class of MHC molecule (thymocytes 2 and 5; the case above of the female TCR transgenic thymocytes recognizing the correct class I allele). Recognition of class I molecules that cross-link TCR with CD8 molecules directs differentiation towards CD8 lineage (Teh et al. 1988; Kisielow et al. 1988b), and recognition of class II molecules that cross-link TCR with CD4 molecule directs differentiation towards CD4 lineages (Kaye et al. 1989): helpers (Th) and suppressors (Treg) (Jordan et al. 2001). Binding antigen by mature T lymphocytes enhance the signal generated by contact with MHC alone and stimulates them to fulfill their duty. CD8 T cells kill non-self and abnormal self cells, while CD4 T cells regulate (enhance or suppress) the activity of B lymphocytes and CD8 T cells.

The mechanism of negative selection of harmful self-antigen reactive lymphocytes is not flawless and some self-reactive T cells (it is not known how many) sneak through. Fortunately, positive selection in addition to helper CD4-Th cells also “educates” inhibitory CD4-Treg cells (Sakaguchi et al. 1982; Sakaguchi 2004), which are able to suppress the activity of autoreactive T cells and also block autoinflammatory responses.

The exploitation of our knowledge of the cellular and molecular mechanisms of lymphocyte development and their regulatory functions for the benefit of patients is a great challenge for medicine.
